# Phenotypic and functional characteristics of monocyte subsets in the blood and bone marrow of Indian subjects with Visceral Leishmaniasis

**DOI:** 10.1371/journal.pntd.0012112

**Published:** 2024-04-26

**Authors:** Gulafsha Kausar, Shashi Bhushan Chauhan, Ritirupa Roy, Vimal Verma, Sundaram Pandey, Aziza Niyaz, Jaya Chakravarty, Christian R. Engwerda, Susanne Nylen, Rajiv Kumar, Mary E. Wilson, Shyam Sundar

**Affiliations:** 1 Department of Medicine, Institute of Medical Sciences, Banaras Hindu University, Varanasi, India; 2 QIMR Berghofer Medical Research Institute, Brisbane, Australia; 3 Department of Microbiology Tumor and Cell Biology, Karolinska Institutet, Stockholm, Sweden; 4 Centre of Experimental Medicine & Surgery, Institute of Medical Sciences, Banaras Hindu University, Varanasi, India; 5 Departments of Internal Medicine and Microbiology & Immunology, University of Iowa and the VA Medical Center, Iowa City, Iowa, United States of America; Universidade Federal de Minas Gerais, BRAZIL

## Abstract

Visceral leishmaniasis (VL) is a potentially fatal parasitic infection caused by *Leishmania donovani* in India. *L*. *donovani* is an obligate intracellular protozoan residing mostly in macrophages of the reticuloendothelial system throughout chronic infection. Monocytic phagocytes are critical in the pathogenesis of different forms of leishmaniasis. Subsets of monocytes are distinguished by their surface markers into CD14+CD16- classical monocytes, CD14+CD16+ intermediate monocytes, and CD16++CD14^low^ non-classical monocyte subsets. During cutaneous leishmaniasis (CL), intermediate monocyte are reported to be a source of inflammatory cytokines IL-1β and TNF, and they express CCR2 attracting them to sites of inflammatory pathology. We examined monocyte subsets in the blood and bone marrow of patients with VL from an endemic site in Bihar, India, and found these contrasted with the roles of monocytes in CL. During VL, intermediate and non-classical CD16+ monocyte subsets expressed instead a non-inflammatory phenotype with low CCR2, high CX3CR1 and low microbicidal oxidant generation, making them more similar to patrolling monocytes than inflammatory cells. Bone marrow CD16+ monocyte subsets expressed a phenotype that might be more similar to the inflammatory subsets of CL, although our inability to obtain bone marrow from healthy donors in the endemic region hampered this interpretation Overall the data suggest that CD16+ intermediate monocyte subsets in VL patients express a phenotypes that contributes to an immunosuppressed pathologic immune state, but in contrast to CL, these do not mediate localized inflammatory responses.

## Author summary

The parasite *Leishmania donovani* causes the fatal systemic disease visceral leishmaniasis (VL) in endemic nations including India. The parasite is an obligate intracellular protozoan, residing mostly in monocytic cells in the bone marrow, blood and affected organs of infected human or other vertebrate hosts. Cells of the monocytic lineage can either provide a safe intracellular location in which the parasite survives, or they can be activated to kill intracellular parasites. We studied the contribution of monocyte subsets in the blood and bone marrow of patients from India with active visceral leishmaniasis, or control subjects without leishmaniasis. Our studies suggested that circulating monocytes of patients with VL are defective in their hability to kill intracellular parasites and to migrate toward sites of inflammation. These defects are most prominent in expanded subsets of monocytes expressing both the CD16 and CD14 receptors on their surface membranes. The immune defects underlying VL may be greatly influenced by subsets of blood monocytes that expand during disease.

## Introduction

Visceral leishmaniasis (VL), also known as kala-azar, is a potentially fatal infection caused in India by the protozoan *Leishmania donovani*. Among the 79 endemic countries, India contributes 28% of the world’s VL burden [1], VL is a chronic infection that has a fatal outcome in 95% of untreated individuals, and a 5–10% mortality even in patients who are treated [2,3]. The causative parasites are obligate intracellular protozoa, most of which reside in tissue macrophages during chronic disease [4]. The outcome of infection is determined largely by host immune responses to the infection, which are most readily studied in humans by examining circulating blood cells. CD8 and CD4 T cells are well-documented critical determinants of host immune responses in leishmaniasis, which can mediate either protective or pathological responses [5–8].

Monocytes show heterogeneity and plasticity in their phenotypes, varying with the local inflammatory microenvironment in host blood or tissues during infection and disease. For instance, circulating monocytes in patients with cutaneous leishmaniasis (CL) due to *L*. *braziliensis* display high levels of TLR2 and TLR4 on their surface and are proposed to mediate inflammatory responses [9,10]. Recent reports have shown that subsets of monocytes which differ in their surface expression levels of the lipopolysaccharide co-receptor CD14 and the FcIIIγa receptor CD16a display different functional characteristics. Three major monocyte subsets (CD14^+^CD16^−^ classical, CD14^+^CD16^+^ intermediate, CD14^low^CD16^++^ non-classical) have been described in humans [11,12]. Classical monocytes are the most abundant, but intermediate and non-classical have been associated with inflammatory conditions [13].

It was recently recognized that subsets of monocytes in the blood participate in the pathogenesis of CL, releasing cytokines that mediate host inflammation and displaying chemokine receptors that guide monocytes to sites of pathological inflammation [9,14]. The roles of the different monocyte subsets in VL have not yet been delineated. In particular, it is not known whether individual monocyte subsets exhibit inflammatory or leishmanicidal responses, and whether they display chemokine receptors influencing their trafficking to tissues of the host. The purpose of the present study, therefore, was to explore the phenotypes and roles of monocyte subsets in patients from an endemic region of India with VL. Because our samples were obtained at the time of initial diagnosis, we were able to study monocyte subsets from peripheral blood and bone marrow of patients with VL. Control blood samples were drawn from endemic control (EC) individuals living in the *L*. *donovani* -endemic site. Control bone marrows were derived from diagnostic bone marrow aspirates done on individuals seen in a hospital in the neighboring state of Uttar Pradesh, India. Our data show there are both phenotypic and functional differences between monocytic cell subsets in infected versus uninfected individuals. These differences likely affect the outcome of infection, and thus might be targets for improved by treatment or preventive measures.

## Methods

### Ethics statement

The guiding principles of the Helsinki Declaration were followed in the enrollment of human subjects. The study protocol was approved by ethical committees of Institute of Medical Sciences, Banaras Hindu University (Dean/2017/EC/185 Dated:24/10/2017), and the Institutional Review Board of the University of Iowa. The BHU IRB was approved by India’s ethical review board and by the US National Institutions of Health. Subject ages ranged between 12–70. Participants or, in the case of children aged 12–17, their legal guardians, supplied written informed permission or assent (age 12–17) at the time of enrollment. VL subjects were recruited prior to treatment. VL patient clinical treatment and follow-up data were kept using standard case report formats on an approved and password-protected server.

The study protocol for propagation of *L*. *donovani* in Syrian hamster was approved by the Central Animal Ethical Committee of the University (Dean/2016/CAEC/699 dated 30.03.2017).

### Study subjects

Patients with VL were diagnosed and treated at the Kala-azar Medical Research Centre (KAMRC) in Muzaffarpur, Bihar, India. VL patients and healthy endemic control subjects were enrolled in the study at KAMRC. Controls for bone marrow aspirates were recruited and entered into the study at Sundar Lal Hospital, which is affiliated with Banaras Hindu University in Varanasi, Uttar Pradesh, India. The latter individuals underwent bone marrow aspirate in diagnosis of a non-infectious condition. The working definition of VL was new onset of signs and symptoms characteristic of VL including fever, fatigue, weight loss, cachexia, and hepatosplenomegaly developing over several weeks, associated with laboratory confirmation of leishmania infection. The latter consisted of a positive reaction to a commercially available serological recombinant K39 (rK39) dipstick strip (InBios Kala Azar Detect Rapid Test, USA), and/or visualization of amastigotes in bone marrow aspirate smears. Endemic control subjects (ECs) were healthy people living in the same village or a village nearby that of a VL patient in the endemic area in Bihar State, India. ECs were tested for rK39, and were confirmed as seronegative before use of their blood for the current studies. ECs were often accompanying family members or friends of VL subjects brought to KAMRC. Controls for bone marrow biopsies were individuals undergoing bone marrow biopsy for diagnosis of non-infectious conditions, who had negative signs, symptoms and peripheral blood serology suggesting leishmaniasis. Exclusion factors for ECs and bone marrow controls included age under 12, pregnancy, previous diagnosis of kala-azar, positive test for HIV, hepatitis, tuberculosis, or malaria, or inability to provide consent. Absence of other medical condition was an additional exclusion criterion for EC subjects. None of the donors had used an anti-inflammatory drug within days before the sample was obtained. Amphotericin B liposomal was administered to all patients with VL after confirmed diagnosis.

The entire study was conducted over a period of 4 years during which multiple trips were made to Muzaffarpur to recruit subjects with VL. Recruitment of patients with VL depended on the numbers of patients admitted to KAMRC at the time when subjects recruited for individual experiments. Considering all subjects entered for the entire study, peripheral blood and/or bone marrow aspirate cells were collected from a total of 20 untreated patients with a confirmed diagnosis of VL, 20 ECs (blood samples), and 20 control subjects undergoing a bone marrow aspirate for diagnoses other than VL. Characteristics of all subjects are summarized in Table 1. No procedures were performed on VL patients or patients providing control bone marrows specifically for the purpose of this research study. Thus, samples were all obtained from leftover cells after a blood draw or a bone marrow aspiration was performed as part of the routine medical care. EC subjects donated peripheral blood samples used for flow cytometry and for CBC and differential counts.

**Table 1:** Summarized characteristics of all Visceral Leishmaniasis (VL) patients, healthy endemic controls, and bone marrow control subjects enrolled in this study. Subsets of these subjects were used for individual experiments, based on the order of their presentation for treatment at KAMRC or Sundar Lal Hospital. Endemic control subjects were enrolled at the same time as VL patients. Numbers of donor cells used in each experiment are indicated in the figure legends. Groups were compared for differences in age (one way ANOVA) or sex distribution (chi square) between the groups.

	VL (n = 20)	Endemic Controls (n = 20)	Bone Marrow Control (n = 20)	p-value
Age (yrs)	36.1 ± 17.9 (13–67)*	35.6 ± 9.7 (27–48)	38.8± 14.5 (20–60)	ns
Sex (F/M)	5/15	4/16	12/8	**ns (VL, EC) 0.0002 (all 3 groups)
Duration of fever (days)	29.3 ± 14.2 (15–60)	NA	NA	
Spleen size (cm below costal margin)	8.8 ± 4.3	0	0	
Bone marrow score ***	1.3± 0.4	NA	NA	

* Mean ± SD (range)

** VL and EC groups did not differ. There was a significant difference in sex ratio between BM control and other groups.

*** Bone marrow score: a microscopic scoring of numbers of parasites in bone marrow aspirates, ranging from 0 (0 parasites/1000 high-power fields) to +6 (>100 parasites/ high-power fields).

### Bone marrow aspirate and whole blood collection

Before beginning VL therapy, diagnostic bone marrow aspirate samples were obtained at the KAMRC from subjects with symptoms suggested of VL. Control bone marrow aspirates were obtained at Sundar Lal Hospital affiliated with Banaras Hindu University, Varanasi. The leftover bone marrow aspirate cells remaining in the needle (~500±250μl, 7±1 million cells) was used for the current analyses. All bone marrow aspirates were performed for clinically indicated diagnostic purposes. After processing the sample for clinical purposes, needles containing remaining cells from the aspirate were flushed into 1 mL of heparinized RPMI 1640 medium (Gibco, USA) with 10% heat-inactivated inactivated (HI)-FBS, 50 units/ml of penicillin, 50 g/ml of streptomycin, and 25 mM HEPES (Gibco). These cells in suspension were transported at 4°C from KAMRC to the Banaras Hindu University lab. Before starting medication, 6 mL of peripheral venous whole blood that had been heparinized was taken from each patient or from endemic healthy controls, and used for isolation of cells for flow cytometry and for complete blood counts and differential leukocyte determinations. Additionally, control bone marrow aspirates were collected from subjects at Sundar Lal Hospital underoing the procedure for diagnosis of non-infectious conditions. Although not available at the time of sample acquisition, the final diagnoses of the bone marrow control subjects were obtained later, after experiments were complete. These are listed in S2 Table.

### Isolation of mononuclear cells from blood and bone marrow

Venous Blood (8–10 mL) was collected into heparinized polypropylene tubes from endemic controls and patients with VL before the start of drug treatment (D-0). Leftover bone marrow aspirate cells (500±250 μl, 7±1million cells) were collected into RPMI medium from patients with VL diagnosed at KAMRC, Muzaffarpur, or from control subjects a Sundar Lal Hospital. Mononuclear cells were isolated from peripheral blood or bone marrow cells by buoyant density using Lymphoprep (StemCell Technologies, Oslo, Norway) as previously described [15,16]. Cell viability was checked using 0.4% (w/v) Trypan blue (Lonza, Walkersville, MD) followed by microscopy. Cell viability was >97% for all cell preparations used in this study.

### Flow cytometry

**Fig 1:**
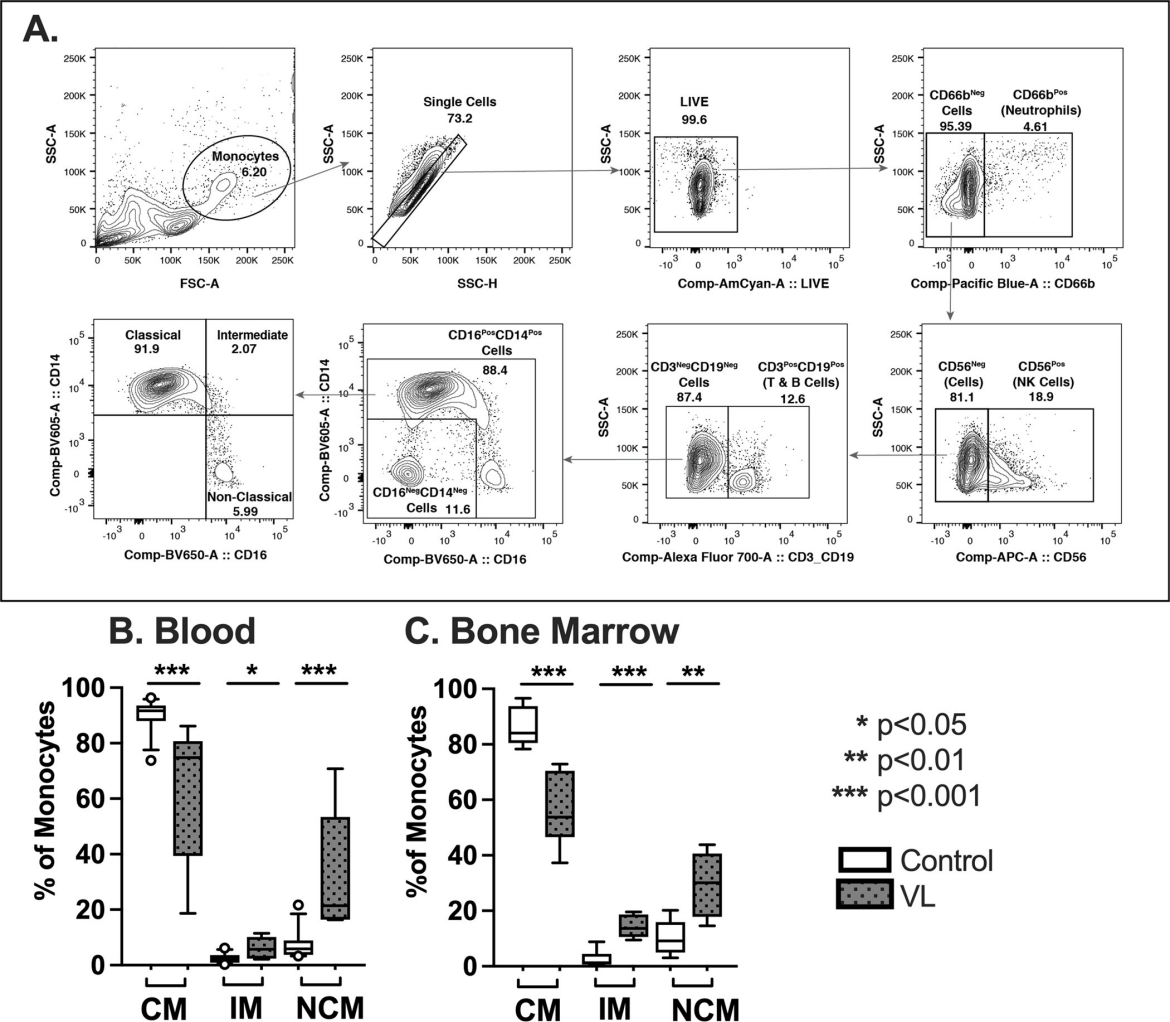
Monocyte subsets in subjects with VL and endemic controls. **(A)** Gating strategy for monocyte subsets. Live, single cells with negative staining for neutrophils, NK cells and lymphocytes were separated into subsets according to CD14 and CD16 surface expression. **(B)** Summary of data for proportions of monocytes belonging to the classical (CM), intermediate monocyte (IM) and non-classical monocyte (NCM) subsets. Shown are whisker plots of the median, upper and lower quartiles, 10% - 90% range and any outlying data points. Controls were either endemic controls (Blood) or bone marrow controls (Bone Marrow) for the indicated data sets. Subjects included 8 VL patients, 12 endemic controls, and 7 bone marrow controls. These subjects included all the consenting patients who presented to KAMRC or BHU medical center at the time the experiment was performed. Statistical comparisons between VL and control data were done using the Mann-Whitney test.

Mononuclear cells from peripheral blood or bone marrow aspirates were suspended in 100ul FACS staining buffer with 2% heat-inactivated fetal bovine serum (FBS; Gibco Thermofisher). Surface antigens were stained with the fluorescently labeled primary antibodies listed in S1 Table. After 30 minutes at 4–8°C, cells were washed and resuspended in 200 μl phosphate-buffered saline/1% (w/v) paraformaldehyde. Data from 30,000 events were acquired using a FACS (Fortessa, BD Biosciences), and analyzed with FlowJo, version 10.8.1 (Tree Star). The gating strategy used for differentiating monocyte subsets is illustrated in Fig 1A.

### Parasites

An isolate of *L*. *donovani* from an Indian patient from KAMRC with documented VL was cultured and maintained by serial intracardiac inoculation in Syrian golden hamsters. Amastigotes were isolated aseptically from hamster spleens 40–60 days after infection [16–18].

### Phagocytosis assay

*L*. *donovani* amastigotes were stained for 20 minutes at 37°C with 5 μM carboxyfluorescein succinimidyl ester (CFSE; catalog number 423801; Biolegend). CFSE-labeled amastigotes and mononuclear cells were incubated at approximately a 3:1 ratio (10^7^ amastigotes, 3.5×10^6^ mononuclear cells) in 100μl phagocytosis buffer (1:1 RPMI: Dulbecco’s modified Eagle’s medium with 1% [w/v] bovine serum albumin and 25 mM HEPES). Following low-speed centrifugation to synchronize phagocytosis, samples were incubated at 37°C, 5% CO_2_. Control mononuclear cells were incubated with parasites at 4°C in phosphate-buffered saline containing 2.5 mM ethylenediaminetetraacetic acid. After 1 hour incubation, monocytes were stained and processed for flow cytometry.

### Detection of reactive oxygen species (ROS)

The CellROX Green flow cytometry kit (catalog number C10492; Invitrogen) was used in accordance with the manufacturer’s instructions to assess ROS generation in monocyte subsets of blood or bone marrow. Briefly, after phagocytosis of amastigotes the CellROX reagent was added to experimental or control cells, and these were returned to 37°C, 5% CO_2_ or 4°C, respectively. Fifteen minutes later the live-dead stain was added, and cells were processed for surface staining by flow cytometry as described above.

### Statistical analyses

Statistical analyses were performed using GraphPad Prism version 8 software (La Jolla, CA, USA) using primarily non-parametric methods. Specific tests are indicated in the figure legends. After correcting for multiple testing, a p<0.05 was used as the cutoff for statistical significance.

## Results

### Comparison of peripheral blood leukocytes in VL Patients versus controls

Patients with documented cases of VL had significantly lower hemoglobin, total leukocyte, and platelet counts before initiating treatment than EC subjects (Table 2). This is not a surprising finding, as pancytopenia is an known characteristic of symptomatic VL [19].

**Table 2:** VL patient and Endemic Control peripheral blood counts (n = 20 in each group).

	VL patients Mean ± SD (range)	Endemic Controls Mean ± SD (range)	p-value (*t*-test)
Hemoglobin (gm /100 ml)	7.8 ± 1.3 (5.8–10.4)	12.1 ± 1.2 (9.4–14.3)	2.49E-13
White blood cells /μl	2,774± 1,549 (1,100–7,600)	6,990 ± 1,080 (5,200–9,000)	5.96E-12
Platelets (platelets /μl)	98,200 ± 38,700 (23,000–208,000)	237,250 ± 82,712 (118,000–384,000)	7.89E-08
Neutrophils (cells /μl)	1,365 ± 760	4,379 ± 896	3.14E-13
Lymphocytes (cells /μl)	1,270 ± 764	2,243 ± 775	4.66E-04
Monocytes (cells /μl)	27.7 ± 15.1	69.9 ± 10.5	5.96E-12
Eosinophils (cells /μl)	110.9 ± 60.3	279.6 ± 42.1	5.96E-12

Because we did not subject healthy individuals to the bone marrow aspiration procedure, control bone marrow aspirates were obtained from persons with other pathologies. The information regarding their diagnoses were not available at the time of the study, but was obtain retrospectively. These diagnoses are listed in S2 Table. The list reveals a spectrum of disorders, ranging from nutrient deficiency and extramedullary hemolysis to blood dyscrasias and acute malignancy. We were not able to link diagnosis with individual data points due to the anonymous nature under which volunteers were recruited. Given this, we did outlier analyses of all studies, and we did not identify any samples for which there were >2 outlying data points. We must, nonetheless, interpret conclusions from studies of bone marrow with caution and acknowledge the limitations.

The proportions of monocyte subsets in subjects with VL and controls were assessed using flow cytometry. Classical, intermediate, or non-classical monocyte subsets were identified according to their expression of surface markers CD14 and CD16, illustrated in Fig 1A. The analyses of collated data showed significant shifts in the proportions of monocyte subsets present in VL patients compared to endemic controls. Both the peripheral blood or the bone marrow of VL patients had significantly lower proportions of classical monocytes, and greater proportions of intermediate and non-classical monocytes, than endemic controls (Fig 1B) or bone marrow controls (Fig 1C). These differences were observed both in the peripheral blood and the bone marrow of subjects with acute VL, each compared to their corresponding controls.

**Fig 2:**
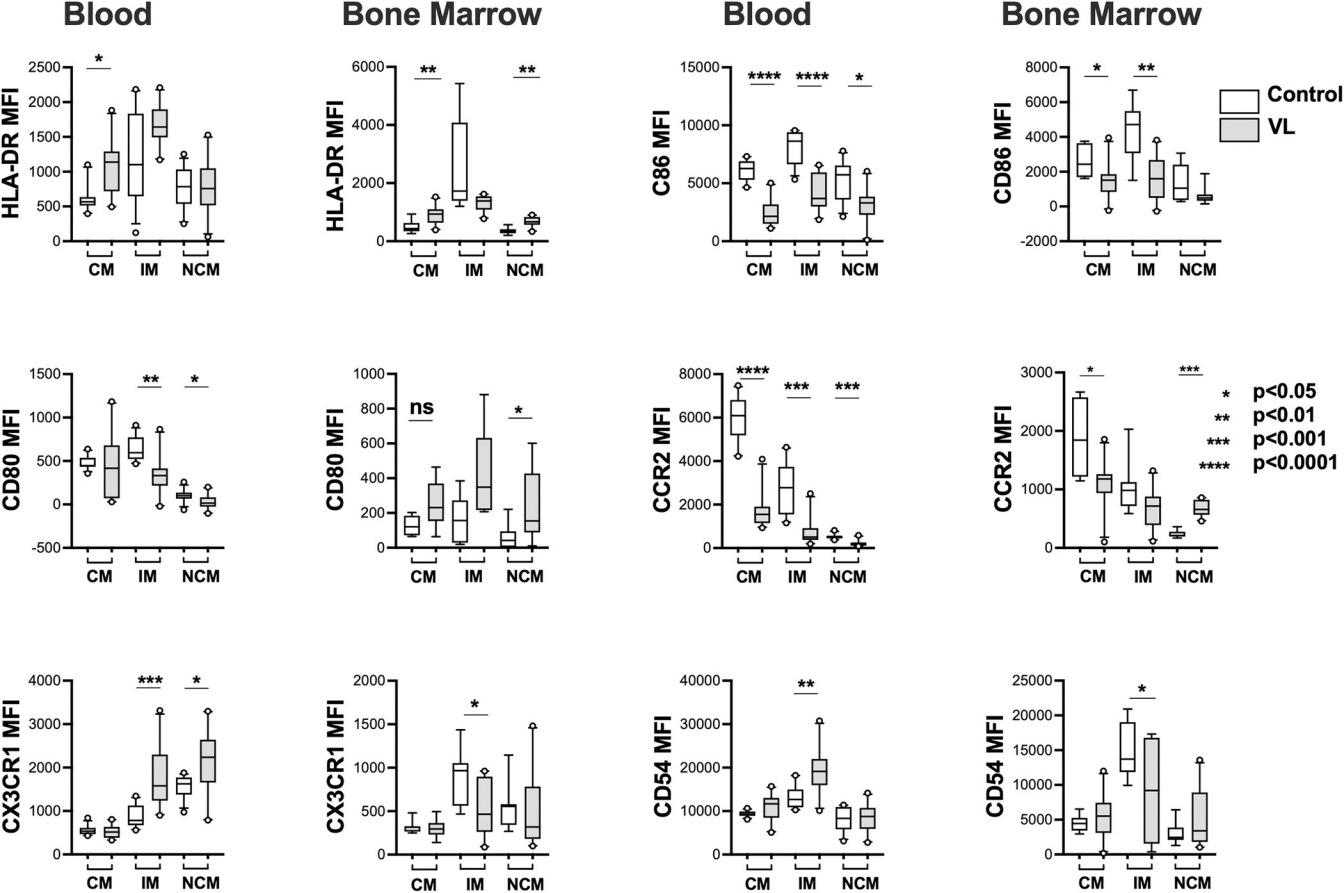
Surface expression of selected markers in monocyte subsets. Expression of surface markers on subsets of monocytes, selected as show in Fig 1, was determined by flow cytometry. Whisker plots show the median, 1^st^ and 3^rd^ quartiles, 10% - 90% range and outer data points. Controls were either endemic controls (Blood) or bone marrow controls (Bone Marrow) for the indicated data sets. Subjects included 10 VL patients, 12 endemic controls, and 7 bone marrow controls. Statistical comparisons between VL and control data were done by Mann-Whitney.

We examined the expression of surface markers that might differ between blood monocyte subsets. Our choices were guided in part by descriptions of Geissmann et al. and others describing a short-lived inflammatory subset of CD14+ monocytes expressing CCR2 enabling them to home to inflammatory sites, but low in CD62L and CX3CR1, versus a “resident” subset high in CD16 and CX3CR1, but low or negative for CCR2 and CD62L [20]. These correspond to the classical / inermediate versus the non-classical subsets. It was subsequently observed that CCR2 is expressed more highly in the intermediate CD14+ monocyte subset during CL, whereas the CCR2-CXCR1+CD16+ monocyte, corresponding to non-classical monocytes, is lower in CL and other inflammatory conditions (9, 21.) We observed that HLA-DR was expressed most highly by intermediate monocytes from both blood and bone marrow, (Fig 2) consistent with its highest expression by CD16+ monocytes in the literature. Similar to our observation with neutrophils [22,23], there was increased surface expression of HLA-DR on classical monocytes in both blood and bone marrow samples from VL patients compared to controls. In contrast, expression of the co-stimulatory molecule CD86 decreased in all subsets of in blood monocytes, and CD80 decreased in intermediate and nonclassical blood subsets. Similarly, CD86 expression was lower on bone marrow monocyte subsets from VL subjects, although CD80 diverged, with higher expression in bone marrow monocyte subsets when compared to bone marrow controls (Fig 2).

Molecules associated with migration into sites of inflammation (CCR2) or adherence to resting endothelium (CX3CR1) also differed between monocyte subsets in VL versus control subjects. Although it remained highest in the classical subset, CCR2 was significantly downregulated in all three subsets of both blood monocytes, with parallel but less dramatic decreased expression by bone marrow monocyte subsets of VL patients. CX3CR1 (fractalkine receptor) in contrast, was upregulated on intermediate and nonclassical blood monocytes compared to controls, but parallel changes were not observed in bone marrow cells. CD54 (ICAM-1) was expressed more highly by intermediate blood monocytes of VL subjects. This marker also trended in the opposite direction in intermediate monocytes from bone marrow cells. If confirmed in other studies, this would imply bone marrow CD16+ monocyte subsets might resemble those of subjects with CL, which are presumed to migrate into sites of inflammation and mediate proinflammatory responses.

### Monocyte subsets from patients with VL show increased phagocytic capacity but reduced generation of ROS

**Fig 3:**
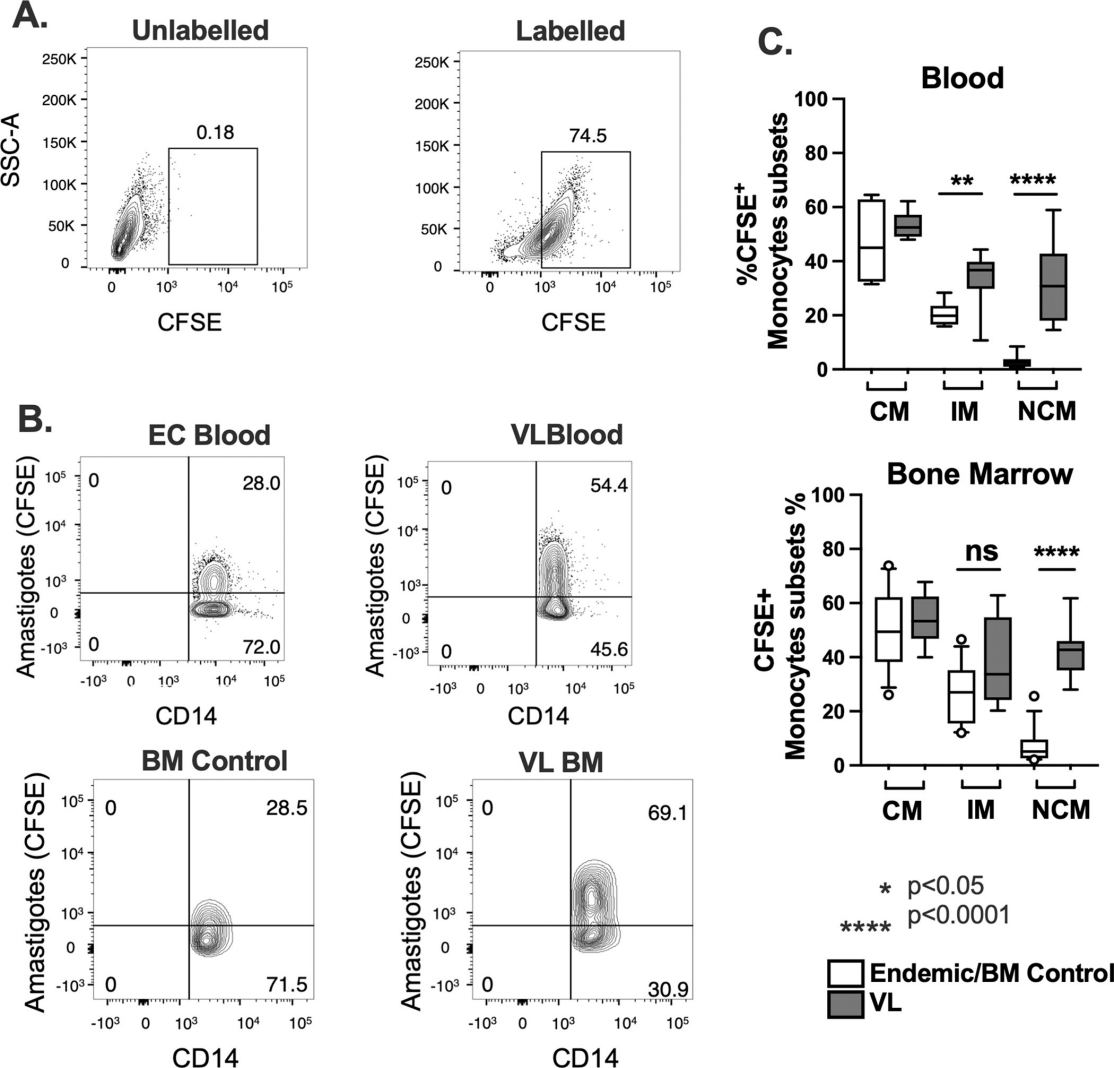
Phagocytosis of *L*. *donovani* by monocyte subsets. *L*. *donovani* amastigotes were labeled by staining with CFSE. **(A)** Side scatter versus fluorescence plots are shown of the amastigotes after CFSE staining. Control amastigotes were stained in parallel conditions, but CFSE was omitted from the stain buffer. **(B** and **C)** CFSE-amastigotes were added at a 3:1 ratio to either PBMCs isolated from peripheral blood of VL subjects or endemic controls, or to bone marrow myeloid cells isolated from either VL or bone marrow control patients. After 1 hour at 37°C, 5% CO_2_, samples were processed for flow cytometry. Shown in **(B)** are representative plots of CFSE stained total CD14+ cells from each subject group, showing the proportions of parasite (CFSE)-positive versus CSFE-negative monocytes. Positive versus negative gates were chosen based on cells in which phagocytosis was inhibited by incubation at 4°C with EDTA. **(C)** Proportions of monocytes in each subset ingesting amastigotes. Box whisker blots show the median, 1^st^ and 3^rd^ quartiles, 10% - 90% range and outer data points for subjects in each phenotype group. Data represent results from 9 patients with VL, 9 endemic controls and 16 control bone marrow patients. Statistical comparisons were generated with the Mann-Whitney test.

**Fig 4:**
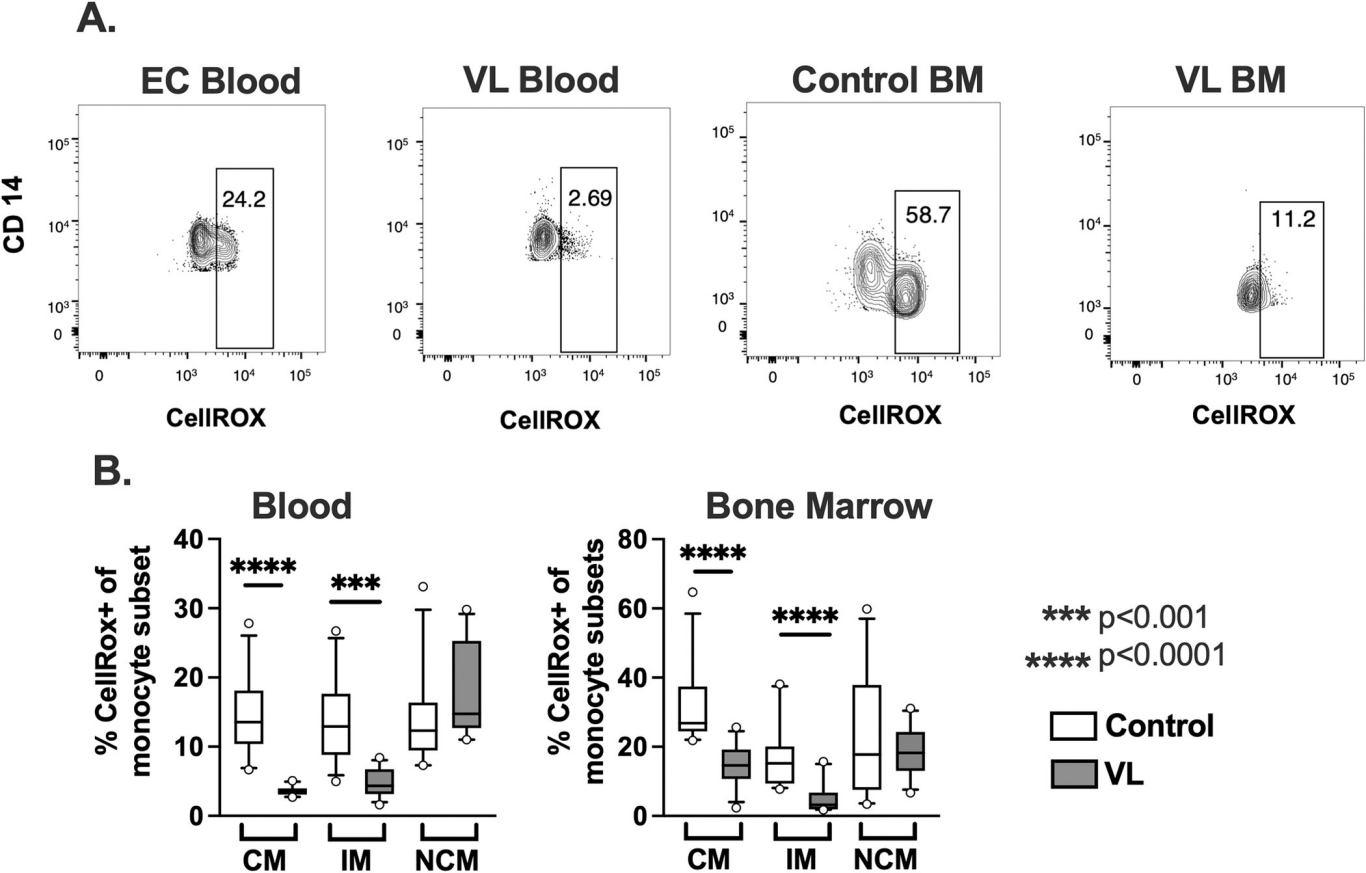
Reactive oxygen species production by monocyte subsets. Mononuclear cells isolated from peripheral blood of VL subjects or endemic controls, or from bone marrows of VL or bone marrow control patients, were infected with *L*. *donovani* amastigotes and incubated at 37°C, 5%CO_2_. The Cell-ROX reagent was added after 15 minutes, live dead stain was added after 30 minutes, and the reaction was stopped after 45 min. Cells were fixed and stained for flow cytometry. **(A)** Representative plots of monocytes gated for CD14 versus CellROX fluorescence. **(B)** Box and whisker plots showing the median, the 1^st^ and 3^rd^ quartiles, 10% - 90% range and outer data points for subjects in each phenotype group. Data represent results from the blood of 12 patients with VL, and 12 endemic controls. Bone marrow myeloid cells were analyzed from 11 of the 12 subjects with VL and 12 bone marrow control patients. Statistical comparisons were generated using the Mann-Whitney test.

We compared functional properties of monocytes from VL patients relative to cells from ECs. The efficiency of parasite phagocytosis was studied by flow cytometry (Fig 3). Amastigotes were labeled *ex vivo* with CFSE and incubated with total mononuclear cell fractions from the blood or bone marrows of different subjects. Cells were stained for surface markers and parasite uptake by classical, intermediate, and non-classical monocyte subsets was assessed by flow cytometry, as illustrated in Fig 3A and 3B. Collated results from all subjects showed that the largest proportion of parasites were taken up by classical monocytes. However, intermediate and non-classical subsets of monocytes from VL subjects had an increased capacity to take up amastigotes compared to control cells of the same subset from either the blood or bone marrow (Fig 3C). In contrast to this increased phagocytosis, generation of ROS, as reflected by oxidation of the CellROX reagent, was significantly decreased in both the classical and intermediate monocyte subsets from VL patients (Fig 4). These differences suggest that monocytes from patients with VL may have suppressed antimicrobial capacity.

## Discussion

The *Leishmania* spp. are obligate intracellular parasites that reside in phagocytic cells, including macrophages, neutrophils, monocytes, and dendritic cells, during this chronic infection [24,25]. The presence of intracellular parasites alters the phenotype and function of infected host phagocytes [26,27]. Leishmaniasis can also alter the phenotype and function of myeloid cells that do not harbor intracellular parasites, through undefined “bystander” mechanisms. For example, the phenotype of uninfected circulating monocytes can reflect the status of localized CL in infected subjects [28,29]. The current study was based on the hypothesis that there are differences between the phenotypes and functions of circulating blood monocyte subsets in patients with VL compared to healthy control individuals living in the same region, and that these differences will reflect the pathogenesis of disease. A corollary is that the disease-unique characteristics of circulating monocytes will reflect the status of monocytes in infected tissues, cells that we presume may be the effectors of disease pathogenesis.

To examine the above hypothesis, we studied peripheral blood monocytes in Indian patients with VL prior to treatment, and compared these to blood of healthy subjects from the same endemic region in the state of Bihar, India. We approached the corollary through examining residual blood cells leftover after diagnostic bone marrow aspirates from VL patients, and compared these to diagnostic bone marrow aspirates from patients with non-infectious conditions.

We previously reported a study of peripheral blood cells from VL patients before and after antiparasitic drug administration. This showed an overall anti-inflammatory phenotype of CD14+ blood monocytes, associated with decreased ability to generate microbicidal molecules but no changes in phagocytosis. In the current report we examined three subsets of monocytes in the blood and bone marrow, distinguished based on their expression of the LPS co-receptor CD14 and the Fc gamma receptor CD16 (FcγRIII) [30]. This analysis enabled us to observe functional and phenotypic differences that were not apparent in the study of total monocytes. Our results showed that subjects with VL had lower proportions of classical monocytes (CD14^hi^CD16-) and higher proportions of intermediate (CD14+CD16+) and nonclassical monocytes (CD14^low^CD16+) compared to healthy endemic control subjects. Similar differences in proportions were observed between bone marrow monocytes of patients with VL compared to patients with non-infectious conditions. The subsets that expanded in patients with VL exhibited both phenotypic and functional differences from the same subsets in control individuals. Both the intermediate and the nonclassical monocyte subsets had increased capacity to internalize amastigotes, but their ability to generate toxic oxidants was impaired. Surface expression of co-stimulatory molecules was low, suggesting intermediate and nonclassical monocytes were defective in their capacity for antigen presentation. Finally, a decreased expression of CCR2 (receptor for monocyte chemoattractant protein-1) but increased CX3R1 (fractalkine receptor) is consistent with decreased attraction towards CCL2 in sites of tissue damage, and a phenotype resembling that of a patrolling monocyte. Overall this resulted in expanded subsets of monocytes in the blood of VL patients that sequester parasites but have arrested capacity to limit their expansion, or to arrest pathological effects. These observations are akin to studies of other inflammatory or infectious conditions, in which a changed phenotype is most prominent in an expanded population of intermediate monocytes [11,31].

Prior studies of the classical, intermediate and nonclassical monocyte subsets in individuals with other conditions document differences between the production of cytokines and chemokines these subsets, and their ability to present antigen [30]. Similar to our observations, HLA-DR (human MHC II) is expressed most abundantly on intermediate (CD14+CD16+) monocytes [30]. Intermediate and non-classical monocyte subsets often expand in conditions of systemic inflammation and infection, and express an inflammatory profile [13,32,33]. A study of humans with CL due to *L*. *braziliensis* were consistent, in that intermediate and nonclassical monocyte subsets were shown to expand [9]. The same subsets also expanded and in subjects with VL in the current study. However the phenotypes of these expanded populations differed between patients with CL versus patients with VL. In the blood of CL patients, the intermediate subset was found to express TNF and IL-1β, both of which are associated with the pathological inflammatory changes that are characteristic of human CL [9,34]. Furthermore, both classical and intermediate monocytes of CL patients expressed higher levels of CCR2, the receptor for MIP-1 (CCL2), whereas non-classical monocytes did not. There was a correspondingly high expression of the ligand CCL2 in lesions, leading the authors to hypothesize that intermediate monocytes contribute toward the pathologic inflammation, and that these inflammatory monocytes are attracted through chemokine gradients to skin lesions [9].

Our findings in the current study of monocyte subset functions in VL were in some aspects the opposite of the CL phenotype. Like other inflammatory conditions, the intermediate monocyte subset in both the blood and bone marrow of subjects with VL expressed the highest levels of HLA-DR [21]. Comparisons between subject groups showed HLA-DR expression in VL blood was further elevated on classical monocytes, but unchanged in the other subsets. However, the expression of co-stimulatory molecules CD80 and CD86 was decreased in blood CD16+ subsets (intermediate and non-classical) in patients with VL, suggesting the capacity of HLA-DR high intermediate monocytes to present antigen may be diminished.

Differences between chemokine receptors in monocyte subsets of patients with VL versus CL paralleled the different immune pathogenesis of these two diseases. Peripheral blood monocytes of all subsets in VL subjects had decreased expression of CCR2, in contrast to the increased monocyte CCR2 observed in monocyes of CL patients [9]. This could indicate that monocyte subsets of VL patients are not prone to migrate into sites of inflammation, in contrast to the CCR2-high intermediate monocytes of CL patients. Expression of surface CX3CR1, which is low in blood of subjects with CL, was elevated on both intermediate and non-classical monocyte subsets of patients with VL. Fractalkine receptor binds to fractalkine on endothelium, and is involved in patrolling of monocytes in uninflamed tissues [20]. These data suggest that, unlike patients with CL, the circulating intermediate and nonclassical monocyte subsets of patients with active VL have impaired ability to migrate into inflammatory tissue sites, and are more likely to remain intravascular. The decreased CCR2, increased CX3CR1 phenotype in VL patients’ intermediate monocytes also differed from other inflammatory conditions [21].

Intermediate and non-classical monocytes from the peripheral blood of patients with VL exhibited increased capacity to take up *L*. *donovani* amastigotes by phagocytosis. Despite this, monocytes from patients with VL produced significantly lower amounts of reactive oxygen species than monocytes of control subjects, suggesting their ability to kill intracellular parasites was impaired. We did not explore the mechanism of this impairment, although possibilities include failure to ligate surface receptors that efficiently activate the NADPH oxidase, active suppression of either cell activation or oxidase assembly, or scavenging intracellular radicals.

One would hypothesize that the phenotype of monocyte subsets in the blood of patients with either VL or CL will reflect even more dramatic differences at the site of parasite residence in infected tissues. Our findings in bone marrow samples did not support this hypothesis. Comparison between bone marrow aspirate cells from patients with VL or patients unndergoing a diagnostic bone marrow aspiration for a non-infectious condition showed that all 3 monocyte subsets expressed significantly higher levels of CD80 than controls. CCR2 was increased on nonclassical monocytes and CXCR1 was decreased on intermediate monocytes, which were both opposite to the patterns in peripheral blood of patients with VL. These data do not suggest impaired inflammation, but are similar to the inflammatory monocyte subsets observed in the blood or at sites of inflammation in humans or murine models with CL [9,20,35]. It is possible that monocyte subsets play different roles in the peripheral blood versus bone marrow during active VL. However, these differences must be regarded with caution, since we were unable to obtain control bone marrow from healthy endemic individuals.

In summary, studies of blood monocyte subsets from subjects with acute leishmaniasis showed that CD16+ intermediate monocytes were expanded and displayed the most dramatic phenotypes in the current study of patients from India with VL, and in published reports of individuals with CL from northeast Brazil. However, the functional capacities of these subsets differed between the different clinical forms of disease. In contrast to CL, CD16+ blood monocytes in patients with VL exhibited a non-inflammatory phenotype with impaired expression of proteins necessary for antigen presentation and migration toward inflammatory sites, and defective microbicidal responses. We hypothesize that CD16+ monocyte subsets in the blood of patients with VL may attenuate host immune responses and enhance survival of parasites, as opposed to their role as mediators of inflammation in CL [34]. Further investigations are needed to show definitively whether these observations are paralleled at sites of parasite growth such as the bone marrow.

## Supporting information

S1 Table Antibodies used for flow cytometry in this manuscript. (DOCX)

S2 Table Final diagnoses of subjects providing bone marrow control samples. (DOCX)
