# Supplementary material for: Phenotypic and functional characteristics of monocyte subsets in the blood and bone marrow of Indian subjects with Visceral Leishmaniasis
Source: PLoS Negl Trop Dis. 2024 Apr 26;18(4):e0012112. doi: 10.1371/journal.pntd.0012112 (PMC11108134; doi:10.1371/journal.pntd.0012112)
Supplement: S1 Table — (DOCX) [file pntd.0012112.s001.docx]

**S1 Table. Antibodies used for flow cytometry in this manuscript**.

| Marker | Clone number | Dye | Company^1^ | Catalogue number |
| --- | --- | --- | --- | --- |
| CD14 | M5E2(RUO) | BV605 | BD | 564054 |
| CD16 | 3G8(RUO) | BV650 | BD | 563692 |
| CD66b | G10F5(RUO) | BV421 | BD | 562940 |
| CD56 | NCAM16.2(RUO) | APC | BD | 341025 |
| HLA-DR | L243(RUO(GMP) | Percp | BD | 347364 |
| CD80 | L307.4(RUO) | PE | BD | 557227 |
| CX3CRI | 2A9-1(RUO) | APC/CY7 | Biolegend | 341616 |
| CCR2 | SA203G11(RUO) | FITC | Biolegend | 357216 |
| CD86 | IT2.2(RUO) | PE/DAZZLE | Biolegend | 305434 |
| CD54 | HA58(RUO) | PECY7 | Biolegend | 353116 |
| Live Dead | N/A^2^ | Zombie Aqua | Biolegend | 423101 |

^1^ BD – Becton Dickinson

^2^ NA – not applicable
