# Supplementary material for: Phenotypic and functional characteristics of monocyte subsets in the blood and bone marrow of Indian subjects with Visceral Leishmaniasis
Source: PLoS Negl Trop Dis. 2024 Apr 26;18(4):e0012112. doi: 10.1371/journal.pntd.0012112 (PMC11108134; doi:10.1371/journal.pntd.0012112)
Supplement: S2 Table — (DOCX) [file pntd.0012112.s002.docx]

**S2 Table. Final diagnoses of subjects providing bone marrow control samples.**

| **Age/Sex** | **Diagnosis** |
| --- | --- |
| 40/F | Nutritional deficiency anemia with Vitamin B12 deficiency |
| 35/M | Nutritional deficiency anemia with Vitamin B12 deficiency |
| 35/F | Myelodysplastic Syndrome |
| 25/F | Iron deficiency with superimposed vitamin B12 deficiency |
| 34/M | Hypercellular Marrow with erythroid hyperplasia |
| 25/F | Hypercellular Marrow/ Hypersplenism |
| 35/M | Acute Leukemia |
| 35/F | Acute Leukemia |
| 15/M | Hemophagocytic lymphohistiocytosis (HLH) |
| 21/M | Hypercellular Marrow |
| 34/M | Hemolytic Anemia |
| 75/M | Hemolytic Anemia |
| 41/F | Iron deficiency anemia |
| 33/F | Chronic Myeloproliferative Disorder |
| 38/F | Essential Thrombocythemia |
| 46/F | Essential Thrombocythemia |
| 45/M | Primary Myelofibrosis |
| 61/M | Plasma Cell Dyscrasia |
| 31/M | Acute Leukemia |
| 46/M | Nutritional deficiency anemia with Vitamin B12 deficiency |
